# The DiSCoVeR trial – Mid-study look at post-training patient motivation for an innovative treatment approach

**DOI:** 10.1192/j.eurpsy.2024.254

**Published:** 2024-08-27

**Authors:** L. Rubene, L. Konošonoka, A. Stūrmane, E. Dechantsreiter, F. Padberg, D. Bavelier, F. Hummel, O. Bonne, Y. Benjamini, M. Nahum, E. Rancāns

**Affiliations:** ^1^Department of Psychiatry and Narcology, Riga Stradiņš University; ^2^Riga Centre of Psychiatry and Addiction Disorders, Riga, Latvia; ^3^Department of Psychiatry and Psychotherapy, LMU University Hospital, Munich, Germany; ^4^Campus Biotech & Faculty of Psychology and Educational Sciences, University of Geneva; ^5^Neuro-X Institute (INX) and Brain Mind Institute (BMI), École Polytechnique Fédérale de Lausanne (EPFL), Geneva, Switzerland; ^6^Department of Psychiatry, Hadassah Medical Center, Faculty of Medicine; ^7^Department of Statistics and Data Science; ^8^School of Occupational Therapy, Faculty of Medicine, The Hebrew University of Jerusalem, Jerusalem, Israel

## Abstract

**Introduction:**

The DiSCoVeR Project: ‘Examining the synergistic effects of a cognitive control videogame and a self-administered non-invasive brain stimulation on alleviating depression’ is a double-blind, sham controlled, randomized controlled trial investigating the feasibility and efficacy of an innovative, self-applied treatment approach for patients diagnosed with major depressive disorder. The trial is conducted at three clinical trial sites (Hadassah, Israel; Riga Stradiņš University, Latvia; Ludwig-Maximilian-University, Germany). The treatment approach combines prefrontal transcranial direct current stimulation with a videogame designed to enhance cognitive and emotional control. This treatment is self-applied at home and remotely monitored. At the beginning of the intervention the patients are randomized in an active group receiving both active stimulation and videogame and the other group receiving sham stimulation and visually similar but not active videogame.

**Objectives:**

The present interims analysis after half of the patients included examines patients’ intrinsic motivation after completing the first five sessions (of 30) of the treatment. We also examine patients’ interest/enjoyment, perceived competence, effort, felt pressure/tension, and perceived choice following the first week of treatment. Intrinsic motivation has been associated with enhanced learning and performance, so it can be used as one of the predictors for patient compliance.

**Methods:**

At the end of the 5^th^ session, the patients filled in the Intrinsic Motivation Inventory (IMI) including the following subscales: interest/enjoyment, perceived choice, perceived competence, effort/importance and felt pressure/tension (scored on a 7-point Likert scale, ranging from 1 “not at all true” to 7 “very true”).

**Results:**

This report includes the first 55 patients randomized (27 patients in the active group and 28 patients in placebo group) for the DiSCoVeR trial. Patients rated their overall interest/enjoyment at 4.50 out of 7 (SD±0.17 95% CI 4.16 to 4.84), their perceived choice at 5.55 (SD±0.16; 95% CI 5.23 to 5.87), their perceived competence at 4.52 (SD±0.15; 95%CI 4.22 to 4.82), their effort/importance at 5.07 (SD±0.16; 95%CI 4.74 to 5.40) and their pressure/tension at 3.00 (SD±0.13; 95% CI 2.73 to 3.26).

**Conclusions:**

We conclude that overall patients were quite interested in the treatment and had inherent pleasure while doing the sessions, felt that it was their choice to do them, felt that they performed the task quite effectively, were invested in doing the sessions and the experienced pressure and tension were low. The perceived choice and competence are positive predictors of intrinsic motivation. This aligns with the previous published data of a smaller patient subset (L. Konosonoka et al Medicina (Kaunas) 2022;58(Supplement 1):72) with the standard deviations being smaller in our larger patient sample.

**Disclosure of Interest:**

None Declared

